# 

**Published:** 1856-01

**Authors:** 


### INDEX.

Abdomen, wound of the 572
Action of medicines on the system, Mr. Headland's work on, notice of 157
Address of Dr. Wood 333
Adulteration of food 45
Agnew, Dr. D. Hayes, case of Epilepsy 213
Agnew, Dr. D. H., Practical Anatomy, a new arrangement of the London Disector, notice of 758
Ague, cure of, by iodide of potassium 447
Alison, pension to Dr. 246
Allen, Dr. J. M., the Practical Anatomist, or Student's Guide in the dissecting room, notice of 757
American Med. Society in Paris, circular of 246
American Med. Association, proceedings of 330
Ammonium, notes on physiological and therapeutical effects of chloride of, by Dr. A. Lindsay 58
Amyloid degeneration, on the course of 380
Analytical Compendium of the Medical Sciences, notice of 242
Anatomical Remembrancer, with additions by C. E. Isaacs, M. D. notice of 117
Arrangement of the muscular subtances of the urinary organs of the human body 560
Apoplexy, &c. a plea for non-performance of marriage 177
Apoplexy during labor 443
Aran, M., on treatment of hæmoptysis 173
Artificial respiration, favorable position for practising 310
Assimilated rank in the navy, letter from a surgeon on 159
Atlas of Cutaneous Diseases, notice of 374

B

Brain, tubercle of, with remarks, Wm. Pepper, M. D. 705
Bamberger, Dr. H., contributions to the physiology and pathology of the heart 720
Bandaging, and other Operations of Minor Surgery, Dr. F. W. Sargent's work on, notice of 24
Barlow, Dr. Geo. Hilaro, Manual of Practice of Medicine, notice of 430
Beck, Dr. John B., Essays on Infant Therapeutics and Lectures on Materia Medica, notice of 700
Becquerel on dropsy of pregnancy 509
Belladonna, effect of in arresting the secretion of milk 567
Bennett's, Dr., Introductory Lecture 164
Bennett, Dr. H., Review of present state of Uterine Pathology, notice of 613
Blackman, Dr. Geo. C., letter from 636
Bowman, John E, Practical Handbook of Medical Chemistry, notice of 304
Bowman, Dr. John E., introduction to practical chemistry, notice of 757
Brachet, J. L., on use of sub-acetate of lead in hypertrophy of the heart 248
Bronzed skin, &c. 316
Brown-Sequard's discovery of the functions of the spinal marrow 187
Brown-Sequard's experimental researches 341
Brown, Dr. Samuel P., case of inversio-uteri 321
Brown, Dr. Isaac B., on diseases (surgical) of women, notice of 374
Budd, Dr. Chas. A., on treatment of prolapsus uteri, by tannin 190
Budd, Dr. Geo., on Diseases and functional disorders of stomach, notice of 149
Buck, Dr. Gurdon, on the internal derangement of the knee-joint 496

C

Cases, notes of, treated in the ophthalmic hospital China, by Jno. G. Kerr, M. D. 727
Calculus, extraction of a large one from the female bladder 51
Cancer of the Pulmonary Artery, by A. Hernher, of Giessen, 45
Cancerous cells, on the formation and propagation of 48
Carson, Dr. Joseph, Synopsis of Lectures, notice of 117
Carpenter, Wm. B., the Microscope and its Revelations, notice of 469

Cephalocele, case of congenital 449
Census of Canada 56
Cesarean section, case of 497
Chambers, Dr. Thos. K., Digestion and its Derangements, notice of 671
Children's Hospital of Phila., Editorial notice of 40
Chloride of ammonium, Dr. Lindsay on the effects of 58
Chloroform, use of in Edinburgh 43
Chloroform in intermittent fever 252
Chloroform, administration of 434
Chloroform, poisoning by, 616
Chloroform, a case of poisoning by 659
Cholera in Lombardy 47
Chorea, its nature and treatment 525
Chorea, a case of rheumatic 581
Churchill, Dr. Fleetwood, Diseases of Infants and Children, notice of 697
Civilization and depopulation 487
Cleveland, Dr. C. H., Pronouncing Medical Lexicon, notice of 117
Clinical Lectures on Surgery, by M. Nelaton. From notes taken by Dr. F. W. Atlee, notice of 27
Cod-liver oil with quinine 125
Condition of vision as a test of the squinting eye 493
Connection between certain forms of Pneumonia and renal disease 437
Consanguinity in marriages, evils of 567
Contribution to the Physiology of sight, notice of 243
Creosote, beneficial effects of, on warty excrescences 49
Cruveilheir, M., on simple ulcer of the stomach 442
Curling, Mr. T. B., Practical Treatise of the Diseases of the Testes, &c. notice of 536

D

DaCosta, appointment of Dr. 163
Dalton, N., chloroform in intermittent fever 252
Death of Dr. Peebles 45
" Dr. Pennypacker 247
" Dr. Warren 435
" Dr. Porter 438
" Wm. Guthrie 438
" Dr. Clutterbuck 438
Death, singular case of, occurring after delivery 454
Deaths in the French army 552
Death subsequent to delivery, a singular case of 583
Dew point, Prof. J. A. Kirkpatrick's observations on 14
Digestion and its Derangement, notice of 671
Digestion of albumen and flesh, 257
Digitalis, effects of, on the generative organs 564
Diseases and functional disorders of the Stomach. By Geo Budd, notice of 149
Diseases of Women admitting of Surgical treatment, notice of 374
Diseases of Infants and Children, notice of 697
Dissector's Manual, &c. notice of 477
Drake, Memoirs of the life and services of Dr. Daniel, notice of 214
Draper, Dr. John W., Human Physiology, notice of 510
Dropsy of pregnancy 509
Dropsy, repeated tappings in 616
Drowned, rules for restoring the 566
Dunglison, Dr. R., New Remedies, notice of 611
Dunglison, Dr. R., Human Physiology, notice of 610
Duration, average of human life 52
Durkee, Dr. S., case of erythema tuberculatum 307

E

Editorial 40, 375, 431, 548
Elbow joint, disease of 555
Entozoa, researches on origin of 501
Epilepsy, case of, for which the patient was trephined 213
Epidemics, Dr. Southwood Smith on 43
Erysipelas of infants 174
Erythema tuberculatum, &c. case of 307
Essays on Infant Therapeutics, notice of 700
Eve, Dr. Paul F., on the history of the ligature applied to the brachio-cephalic artery 475
Excision of the cervix uteri, for corroding ulcer 140
Experimental researches of Brown Sequard 311
Experiments upon digestion 385, 513

F

Fearing, Dr. E. P., on the extraction of needles, &c. 560
Flint, Dr. Austin, Physical Exploration and Diagnosis of Diseases, &c. notice of 292
Foote, Mr. John, Practitioner's Pharmacopeia, by, notice of 117
Formic acid, formation of, from carbonic acid 329
Four children at one birth 480
Fracture of cervix femoris 126

G

Gardner, Dr. Aug. K., on Causes of Sterility, notice of 427
Gemmill, Dr. J. M., case of death twenty-two days after delivery 454
Gibbs, Dr. O. C., chorea, its nature and treatment 525
Gibbs, Dr. O. C., a singular case of death subsequent to delivery 583
Glycerine, use of in preserving organic bodies 616
Graduates of the Medical Colleges of the U. S., number of 305

H

Hæmoptysis, on treatment of 173
Hall, Dr. Marshall, rules for restoring the drowned 566
Hamilton, Dr. F. Hastings, Report on Deformities after Fractures, notice of 105
Dr. R. P. Harris's letters from Paris 10, 70
Harris, Dr. Wm. cases by 65
Hartshorne, Dr. H., case of rheumatic chorea 581
Hæmorrhage in fevers 185
Heart, contributions to the physiology and pathology of the, by Dr. H. Bamberger, notice of 722
Headland, Mr. F. H., work on the action of medicines 157
Headaches and their Causes, notice of 477
Health of Philadelphia 759
Henry, Dr. Bernard, on idiopathic gangrene of the four extremities 127
Hernia, case of femoral, incarcerated fourteen days 144
Hervieux on erysipelas of infants 174
Hernia, some points in the surgery of 183
Hilgard's contributions to the Physiology of sight, notice of 243
History of medicine. Dr. P. V. Renouard's, Translated by Prof. C. G. Comegy's, notice of 19
History of Ligature applied to the brachio-cephalic artery, notice of 475
Holthouse, C., on condition of vission the best test for the squinting eye 493
Homœopathy, overdose of 255
Homœopathy 569
Hooper on physiological errors of teetotalism 175
Hooping cough, pathology of 507
Horsley, John, on cod-liver oil with quinine 125
Hospital for children 40
" for incurables 43
" for consumption 44
How to nurse sick children, notice of 158
Human Physiology, Statistical and Dynamical, notice of 590
Human Physiology, notice of 610
Hunt, Dr. Sanford B., Report on the Hygrometrical state of the Atmosphere, notice of 301
Huss, Dr. Magnus, Statistics and Treatment of Typhus and Typhoid Fever, notice of 370

I

Idiopathic gangrene of the four extremities, case of, by Dr. B. Henry 129
Imperforate anus, case of 67
Incurables, Royal Hospital for 43
Induction of labor at the seventh month for the relief of obstinate vomiting 65
Insanity and idiocy in Massachusetts, report on, notice of 154
Intermittent fever, cases of 518
Intermittent fever, history of three cases of 577
Inflammation of the os and cervix uteri 167
Inversio-uteri, case of 321
Itch, cure of, in half an hour 447

J

Jackson, Dr. Jas., Letters to a young physician 241
Jewell, Dr. Wilson, on the mortality of Philadelphia; for Oct., Nov. and Dec., 1855, 75; Jan., Feb. and March, 1856, 276; April, May and June 459; July, Aug. and Sept. 661
Jones, Dr. Joseph, on digestion of albumen and flesh and the comparative anatomy and physiology of the pancreas 257
Jones, F. Wharton, Principles and Practice of Ophthalmic medicine, &c. notice 363
Jordan, Mr. T. F., on operation for phymosis 171

K

Kennedy, Dr. Henry, on hæmorrhage in fevers 185
Kerr, Dr. John G., notes of cases treated in the ophthalmic hospital, China 720
Kesteven, on trial for breach of marriage, on plea of paralysis 177
King, appointment of Dr. 244
Kirkpatrick, Prof. J. A., on the dew point 14
Kirkpatrick, Prof. J. A., meteorological observations 64, 127, 172, 256, 320, 384, 448, 512, 576, 640, 704 760

Kirkbride, report of Dr. 245
Knee joint, internal derangement of the 496

L

Landolfi's treatment of cancer 562
Law, Dr. R., observations on pericarditis 625
Lawrie, Dr. J. A., on poisoning by strychnine, with experiments, &c 627
Lectures on materia medica, notice of 700
Lehmann's Physiological Chemistry, notice of 87
Lehmann's Manual of Chemical Physiology, notice of 420
Letter from Paris, by Dr. R. P. Harris 10, 70
Letters to a Young Physician, notice of 241
Levergood, Dr. J., case of triplets 69
Lexicon, Dr. Cleveland's pronouncing medical, notice of 117
Levick, appointment of Dr. James 244
Lewis, Dr. F. W., cases of œdema of a single lower extremity 649
Littell, Dr. S., on malaria not the cause of fever 1
Louisville Review 431
Lyman, Dr. G. H., on the history of ovariotomy, notice of 743

M

Malaria not the cause of fever, by S. Littell, M. D. 1
Manual of Chemical Physiology, notice of 420
Manual of the Practice of Medicine, notice of 430
Mason, Dr. J. K., case of bloody tumor of the vagina, occurring during labor 137
McClintock, Dr. Alf. K., on phlebitis of the great veins of the neck, &c. 619
McClellan, Dr. J. H. B., case of femoral hernia 144
McKinley, prolonged vitality of small-pox contagion 585
McDowel, on connection between certain forms of pneumonia and renal disease 437
Medical Profession in ancient times, notice of 414
Medical Jurisprudence, notice of 542
Meigs, Dr. John, history of three cases of intermittent fever 577
Meigs, Dr. John, a case of pneumothorax, recovery 64
Meigs, Dr. Charles D., on obstetrics; the science and the art, notice of 756
Memoirs of the life and services of of Daniel Drake, M. D., notice of 214
Mesmeric effects 380
Meteorological Observations, for Nov., 1855, 64; Dec., 1855, 127; Jan., 1856, 192; Feb. 256; March 320; April 384; May 448; June 512; July 576; Aug. 649; Sept. 704; Oct. 760
Microscope and its Revelations, notice of 469
Miller, James E., Principles of Surgery, notice of 305
Mills, Dr. Charles, case of Cæsarean section 496
Mitchell, Dr. J. K., tribute of respect from St. Andrew's Society to 42
Moleschott, Dr. J., on the secretion of sugar and bile in the liver 176
Mortality of Philadelphia for Oct. Nov., and Dec., 1855, 75; Jan., Feb. and March, 1856, 276; April, May and June 459; July, Aug. and Sept. 661
Mott, Dr. Valentine, operations by 552
Mussey, Dr. Wm. H., on poisoning by opium, treatment with belladonna 189
Mütter, resignation of Dr. 377

N

Needles, extraction of, from the human body 560
Neill & Smith's Analytical Compendium, notice of 242
Nelaton's Clinical Lectures on Surgery. From Dr. W. F. Atlee's notes, notice of 27
Neligan's, Dr. J. Moore, Atlas of cutaneous diseases 374
Nelken, Dr. M., Sea sickness, its cause, nature, symptoms, treatment, &c. notice of 758
New York, Transactions of the State Society, notice of 473
New York and Philadelphia 549
Neuralgia, treatment of by valerianate of ammonia 587
New Elements of Operative Surgery, notice of 699
Nitrate of silver as a remedy for burns 144
Nocturnal erections, treatment of 564
Nose, congenital absence of 446

O

Obstetric Memoirs and Contributions of James Y. Simpson, M. D. notice of 286, 754
Obstetric tables, notice of 612
Œdema of a single lower extremity, cases of 649
Osteological memoirs, notice of 546
Ovariotomy, history and statistics of, and the circumstances under which the operation may be regarded as safe and expedient, by G. H. Lyman, M. D., notice of 742

P

Palmer, case of Walter 179, 480, 486
Parrish's Introduction to Practical Pharmacy, notice of 38
Peck, Dr. W. A., case of congenital cephalocele 449
Peck, Dr. W. A., cases of intermittent fever 518
Pennypacker, death of Dr. Isaac A. 247
Pennsylvania State Medical Soc., proceedings of 394
Pepper, Dr. W., on tubercle of brain 705
Pericarditis, observations on 625
Periodicals, number of Medical in Europe 702
Phlebitis of the great venous trunks of the neck, subsequent to labor 619
Phymosis, simplest operation for uncomplicated, congenital 171
Physical Exploration and Diagnosis of Disease, notice of 292
Physiological Chemistry. Prof. C. G. Lehmann's, notice of 87
Placenta Prævia, Dr. Trask's prize essay on statistics of, notice of 115
Poisoning by opium, treated with belladonna, case of 189
Poisoning by lobelia inflata 551
Pneumothorax, a case of, with recovery 641
Potassa fusa, use of in boils and carbuncles 124
Practical Pharmacy, Mr. E. Parrish's Introduction to, notice of 38
Practical Handbook of Medical Chemistry, notice of 304
Practitioner's Pharmacopœia, Mr. Foote's, notice of 116
Practical Treatise on Diseases of the Testes, &c. notice of 536
Pregnancy, extensive injuries during 701
Presentation of dead children, on the 253
Principles and Practice of Ophthalmic Medicine, &c. notice of 363
Principles of Surgery, notice of 305
Prolapsus uteri, treatment of by tannin 190
Puerperal Peritonitis, twenty-seven cases of, by Dr. R. K. Smith 193
Puerperal peritonitis, seven cases of, by Dr. Robert K. Smith 709
Pulmonary artery, cancer of 45
Purification of water supplied to towns 503

R

Ramsbotham's cases of spontaneous restoration of the retroverted gravid uterus 122
Reduction of old luxation, fatal 636
Registrar General, Fourteenth Annual Report of the English 378
Remedies, new, notice of 611
Renouard's History of Medicine. Translated by C. G. Comegys, M. D., notice of 19
Review of the present state of uterine pathology, notice of 613
Revolution among the Eclectics 435
Rigby, Dr., on inflammation of the os and cervix uteri 167
Rigid anchylosed human skeleton, case of 326
Robards, Dr., on wounds of the abdomen 572

S

Sargent, Dr. F. W., on bandaging, and other operations of minor surgery, notice of 24
Scarlet fever, coincidence of, with variola 118
Secretion of sugar and bile in the liver 176
Shippen, Mr. Ed., case of Tracheotomy for the removal of a grain of corn 457
Simpson, Dr. James Y., Obstetric Memoirs, &c, notice of 286, 754
Small-pox inoculations 551
Small-pox contagion, prolonged vitality of 585
Smith, Dr. Southwood, on epidemics 43
Smith, Dr. R. K., report of twenty-seven cases of puerperal peritonitis 193
Smith, Dr. R. K., report of seven cases of puerperal peritonitis 709
Smith, Dr. Robert Wm., on Fractures, &c. notice of 237
Smith, Dr. Tyler, action of potassa fusa and cautery on os uteri 314
Smith, Dr. F. G., experiments upno digestion 385, 513

Smith, Dr. Henry, on post-mortem appearances of tracheotomy 558
Smith, Dr. Henry H., treatise on the practice of Surgery, notice of 747
Spontaneous restoration of the retroverted gravid uterus, case of 122
Struthers, Dr. John, Osteological Memoirs, notice of 546
St. Andrew's Society, tribute of respect to Dr. J. K. Mitchell from 42
Statistics and Treatment of Typhus and Typhoid Fever, notice of 370
Sterility, causes and curative treatment of, notice of 427
Stomach, gun-shot wound of 488
Strychnia, tests for 485
Strychnine, poisoning by, with experiments on 627
Subacetate of lead, use of in hypertrophy of the heart 248
Supra-renal capsules and bronzed skin 316
Surgery, a treatise on the practice of, by Dr. H. H. Smith, notice of 747
Sydenham Society, publications of 615
Symptomatic vomiting of pregnancy 119
Synopsis of Lectures on Materia Medica, by Joseph Carson, M.D. notice of 117

T

Tape worm, in connection with the eating of raw pork 253
Taylor, Dr. A. S., Medical Jurisprudence, notice of 542
Teetotalism, physiological errors of 175
Thompson, Dr. Allen, researches on the origin of entozoa 501
Tracheotomy for the removal of a grain of corn 457
Tracheotomy, post-mortem appearances of 558
Treatise on Fractures, &c. notice of 237
Transactions of the Am. Med. Association, notice of 105, 301, 758
Trask's Dr., essay on statistics of placenta prævia, notice of 115
Travers, Benjamin, on the use of potassa fussa, in boils and carbuncles 124
Therapeutics and Pharmacology, or materia medica, treatise on, by George B. Wood, M. D., notice of 728
Triplets, Dr. Levergood's case of 69, 328
Tripler, Dr. Charles S., case of gun-shot wound of stomach 488
Tumor, bloody, of the vagina, occurring during labor 137
Turnbull, Dr. L., on the excision of the os uteri 140
Twins, results of some physiological and statistical researches on 146
Typhus in the Crimea 574

U

Ulcer, simple, of the stomach 442

V

Van der Kolk, Schroder, on formation and propagation of cancerous cells 48
Velpeau, Prof. Alf. A. L. M., New Elements of Operative Surgery, notice of 699
Virchow, Rudolph, on course of the amyloid degeneration 380
Vocation of the medical scholar 445

W

Warty excrescences, beneficial effects of creasote on 49
Warren, death of Dr. 435
Watson, Dr. John, the Medical Profession in Ancient Times, notice of 414
Williams, Dr. Junius, a case poisoning by chloroform 659
Wilson, Dr. H. P. C., case of anchylosis 326
Wilson, Erasmus, Dissector's Manual, notice of 477
Wiltbank, Dr. John, on nitrate of silver in burns 144
Wood, address of Dr. Geo. B., to the Am. Med. Asso. 333
Wood, Dr. George B., treatise on therapeutics and pharmacology, or materia medica, notice of 728
Wright, Dr. Henry G., Headaches and their causes, notice of 477

